# Development and application of a triplex real-time PCR method for the detection of *Lawsonia intracellularis*, *Brachyspira hyodysenteriae*, and *Clostridium perfringens*

**DOI:** 10.1128/spectrum.03237-24

**Published:** 2025-05-14

**Authors:** Wenqing Wu, Lei Wang, Rui Xie, Changjiang Peng, Hao Yang, Hechao Zhu, Yajuan Luo, Lin Hua, Huanchun Chen, Bin Wu, Zhong Peng

**Affiliations:** 1 National Key Laboratory of Agricultural Microbiology, College of Veterinary Medicine, Huazhong Agricultural University 47895, Wuhan, China; 2 Hubei Hongshan Laboratory, Wuhan, China; 3 Frontiers Science Center for Animal Breeding and Sustainable Production, The Cooperative Innovation Center for Sustainable Pig Production, Wuhan, China; 4 Guangxi Yangxiang Co., Ltd., Guigang, China; University of Edinburgh, Midlothian, United Kingdom

**Keywords:** triplex real-time PCR method, *Lawsonia intracellularis *(LI), *Brachyspira hyodysenteriae *(Bhy), *Clostridium perfringens *(Cp), clinical detection, bacterial diarrhea diagnosis

## Abstract

*Lawsonia intracellularis* (LI), *Brachyspira hyodysenteriae* (Bhy), and *Clostridium perfringens* (Cp) are three common swine enteric pathogens widespread worldwide, leading to huge economic losses to the pig industry. LI, Bhy, and Cp are co-infected and should not be distinguished, and the detection of bacterial nucleic acids in feces allows rapid identification of whether a herd is infected with these three pathogens. In this study, we developed and optimized a TaqMan triple quantitative real-time PCR (qPCR) method for LI, Bhy, and Cp, which was validated and applied to the analysis of actual samples from pigs. This method was then applied to investigate 5,702 fecal swabs, environmental swabs, and ileum in China from 2021 to 2024. The results showed that the positive rates of the three pathogens were 9.54% (544/5,702; LI), 2.42% (138/5,702; Bhy), and 20.78% (1,185/5,702; Cp), respectively. In addition, mixed infections between some pathogens were also found.

**IMPORTANCE:**

In this study, we developed a triple real-time PCR method for the detection of *Lawsonia intracellularis* (LI), *Brachyspira hyodysenteriae* (Bhy), and *Clostridium perfringens* (Cp). A total of 5,702 samples from pig farms in China from 2021 to 2024 were detected by this method. The results showed that all three pathogens were detected and mixed infections occurred. This method is rapid, specific, and sensitive and can be used for the monitoring and rapid diagnosis of bacterial diarrhea in pig herds.

## INTRODUCTION

Bacterial diarrhea has a significant impact on the pig industry worldwide, and common pathogens in pig production currently include *Lawsonia intracellularis* (LI), *Brachyspira hyodysenteriae* (Bhy), *Clostridium perfringen*s (Cp), *Salmonella,* and *Escherichia coli* (1–3). LI, Bhy, and Cp can cause hematochezia, acute death, necrotizing enteritis, loss of optimal performance, and reduced daily gain in pigs (4–7). So far, three pathogens have been reported to be prevalent in major pig-raising countries in the world and have been found in herds of all ages (6, 8). Due to similar symptoms and the emergence of mixed infection cases, it is urgent to develop a detection method that can rapidly monitor the three pathogens and provide a basis for guiding the medication of pig farms (9–11). Although pathogen isolation is considered the reference standard for confirmation of infection, it has limitations in sensitivity, duration, technical equipment, and expertise, making rapid diagnosis impractical. Without a conventional bacterial culture medium (12, 13), the isolation and cultivation of the three pathogens are difficult, and rapid detection cannot be achieved, causing great obstacles to the study of the pathogenesis of the disease and prevention and control techniques (14–16). They often co-infect swine, and their clinical symptoms and pathological changes are similar, making it challenging to differentiate between them. Infections with a single pathogen may not always result in symptoms, but complex infections involving multiple pathogens may result in serious illness (6, 10). The detection results combined with specific clinical symptoms, pathological examination, and epidemiological data can provide an important reference for the prevention and control of the disease. The current trend in establishing detection methods is to target multiple real-time PCR, which can detect multiple pathogens in a single reaction system, improve detection efficiency, and save manpower and financial resources (17, 18). The real-time PCR combines PCR amplification and electrophoresis determination into one step, thus saving time and manpower and reducing the risk of residual contamination (19, 20). So far, there have been no reports of qPCR methods for the simultaneous detection of the above-mentioned three pathogens.

The aim of this study was to establish a triple TaqMan real-time PCR assay for rapid, accurate, and sensitive detection of LI, Bhy, and Cp. This method will provide a reliable tool for epidemic disease diagnosis and pathogen detection and provide a scientific basis for formulating corresponding prevention and control measures.

## RESULTS

### Optimization of the amplification conditions

**Fig 1:**
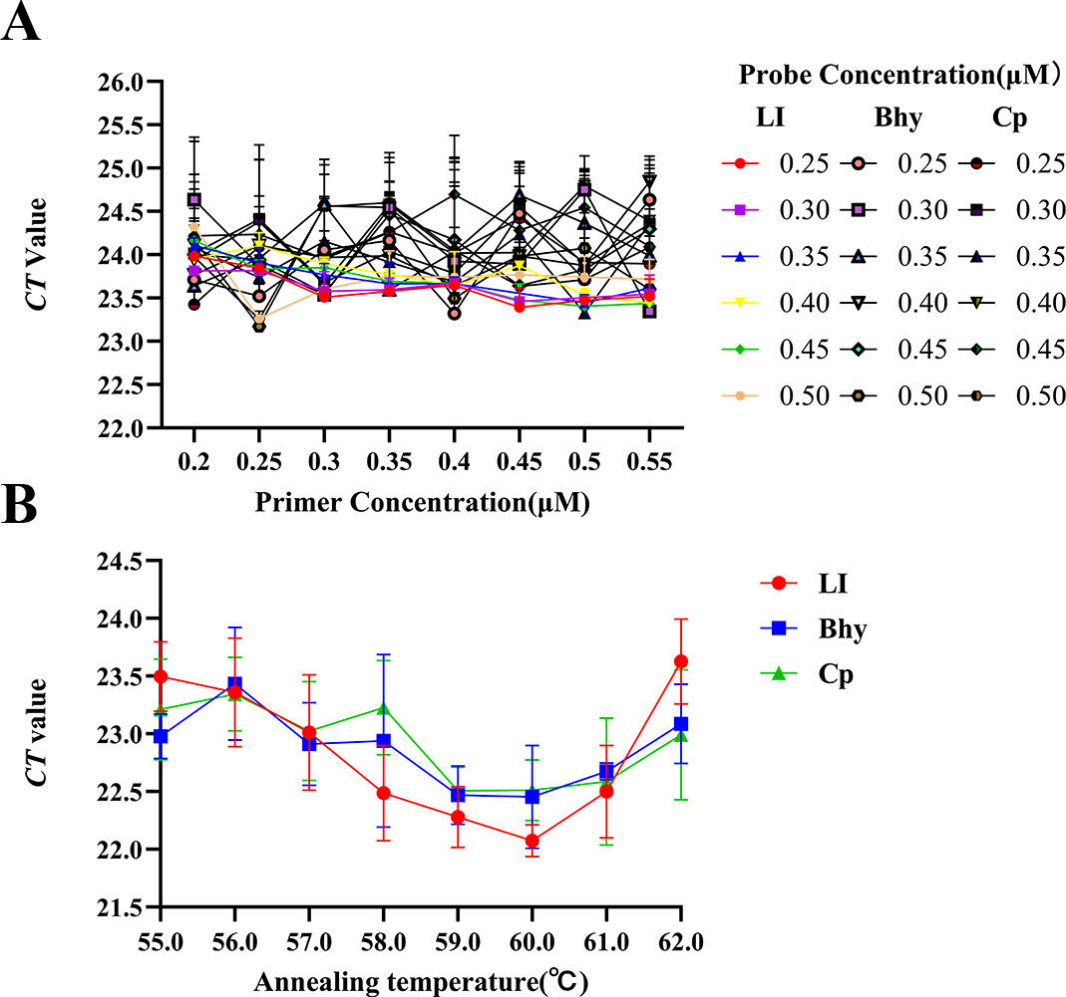
Optimization of the triplex real-time PCR method developed in this study. (**A**) Optimization of probe primer concentration; (**B**) optimization of reaction temperature.

After optimized design of primers and probes (Table 1), we successfully constructed standard plasmids pUC57-LI (100 ng/µL, 2.82 × 10^9^ copies/μL), pUC57-Bhy (100 ng/µL, 5.63 × 10^9^ copies/μL), and pUC57-Cp (100 ng/µL, 1.41 × 10^9^ copies/μL) for three pathogens. We investigated the optimal concentrations of the primers and probes. We detected 10^4^ copies/μL of plasmids with different concentrations of primers (0.20, 0.25, 0.30, 0.35, 0.40, 0.45, 0.50, or 0.55 µM) and probes (0.25, 0.30, 0.35, 0.40, 0.45, or 0.50 µM). To explore the optimal annealing temperature for tr-PCR, we detected the plasmids at 55.0°C, 56.0°C, 57.0°C, 58.0°C, 59.0°C, 60.0°C, 61.0°C, and 62.0°C. The results showed that when the primer concentration was set at 0.25 µM and the probe concentration was set at 0.50 µM, the cycle threshold (Ct) values of the three fluorescence signals were the lowest (Fig. 1A). When the annealing temperature was set at 60°C, the Ct values of the three fluorescence signals were the lowest. Considering the convenience of economic and practical operation, as well as the optimal amplification effect achieved, we determined that the optimal primer concentration was 0.25 µM, the optimal probe concentration was 0.50 µM, and the annealing temperature of the reaction was 60°C as the reaction conditions for the tr-PCR (Fig. 1B).

**TABLE 1:** Primers and probes used in the triplex real-time PCR assay

Primers/probe	Sequences (5′−3′)	Target gene	Size (bp)
LI-F	CCTTGGAGGTAAATTGATTTCTCC	*aspA*	122
LI-R	ATGTTCAGCTTTCTGGTGTTCTTA		
LI-Probe	FAM-TCCACAGCGAGGACCACTTGAGA-TAMRA		
Bhy-F	TATTCGGGCAACTGGATAAG	*16S rRNA*	117
Bhy-R	TGTAGCAAGAGTATATGCGG		
Bhy-Probe	Cy5-AGTCCATGTTTCCATGGGTTATCCCCCA-BHQ2		
Cp-F	TAGCTTAATTTCGGGACTGC	*α-toxin*	99
Cp-R	AGTGATGGAAGACCAACTAA		
Cp-Probe	VIC-ACCTGCAACAGGTACTGAAATAAC-BHQ1		

^*a*^ F, R, and P indicate forward primer, reverse primer, and probe, respectively.

### Detection limit and standard curves

**Fig 2:**
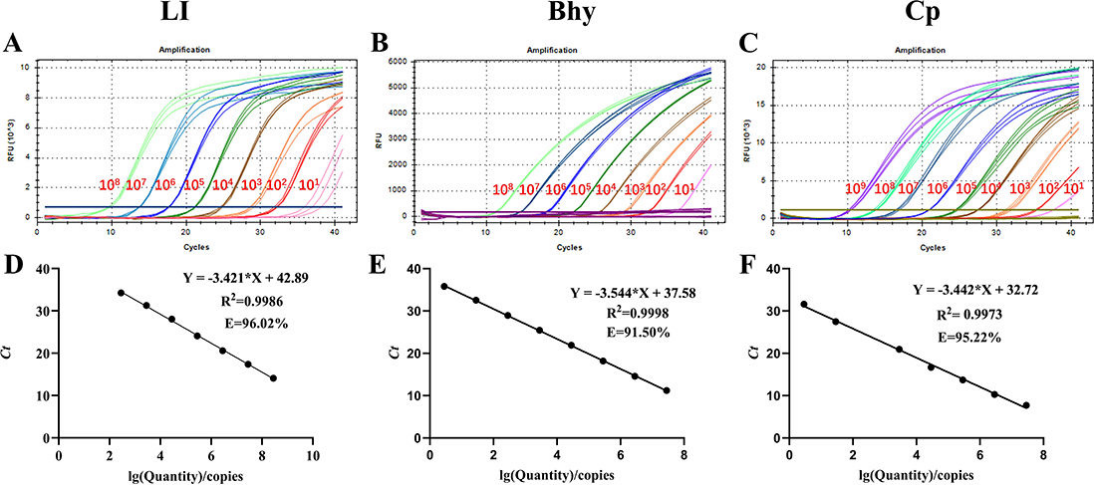
Detection limit and standard curves of the triplex real-time PCR method developed in this study. (**A**) Detection limit for LI; (**B**) detection limit for Bhy; (**C**) detection limit for Cp; (**D**) plasmid DNA standard curve for LI, Y = −3.421x + 42.89, *R^2^* = 0.9986, E = 96.02%; (**E**) plasmid DNA standard curve for Bhy, Y = −3.544x + 37.58, *R^2^* = 0.9998, E = 91.05%; (**F**) plasmid DNA standard curve for Cp, Y = −3.442x + 32.72, *R^2^* = 0.9973, E = 95.22%.

Serial 10-fold dilutions of pUC57-LI (from 2.82 × 10^9^ to 2.82 × 10^−1^ copies/μL), pUC57-Bhy (from 1.41 × 10^9^ to 1.41 copies/μL), and pUC57-Cp (from 5.63 × 10^9^ to 5.63 × 10^−1^ copies/μL) were performed to detect standard plasmids with different copy numbers using the tr-PCR method. Plasmids with different copy numbers were detected using tr-PCR, and the detection limits for LI, Bhy, and Cp are shown in Fig. 2A through C. The assay results were subsequently used to plot a standard curve with GraphPad Prism v.8.0.1, and there was a strong linear correlation (R^2^ > 0.99) between Ct values and corresponding copy numbers for pUC57 LI, pUC57 Bhy, and pUC57-Cp. The slopes of the standard curves for the three standard plasmids were −3.421, –3.544, and −3.442, respectively. Reaction efficiencies were 96.02%, 91.50%, and 95.22% (Fig. 2D through F).

### Stability, specificity, and sensitivity of the triplex real-time PCR method

**Fig 3:**
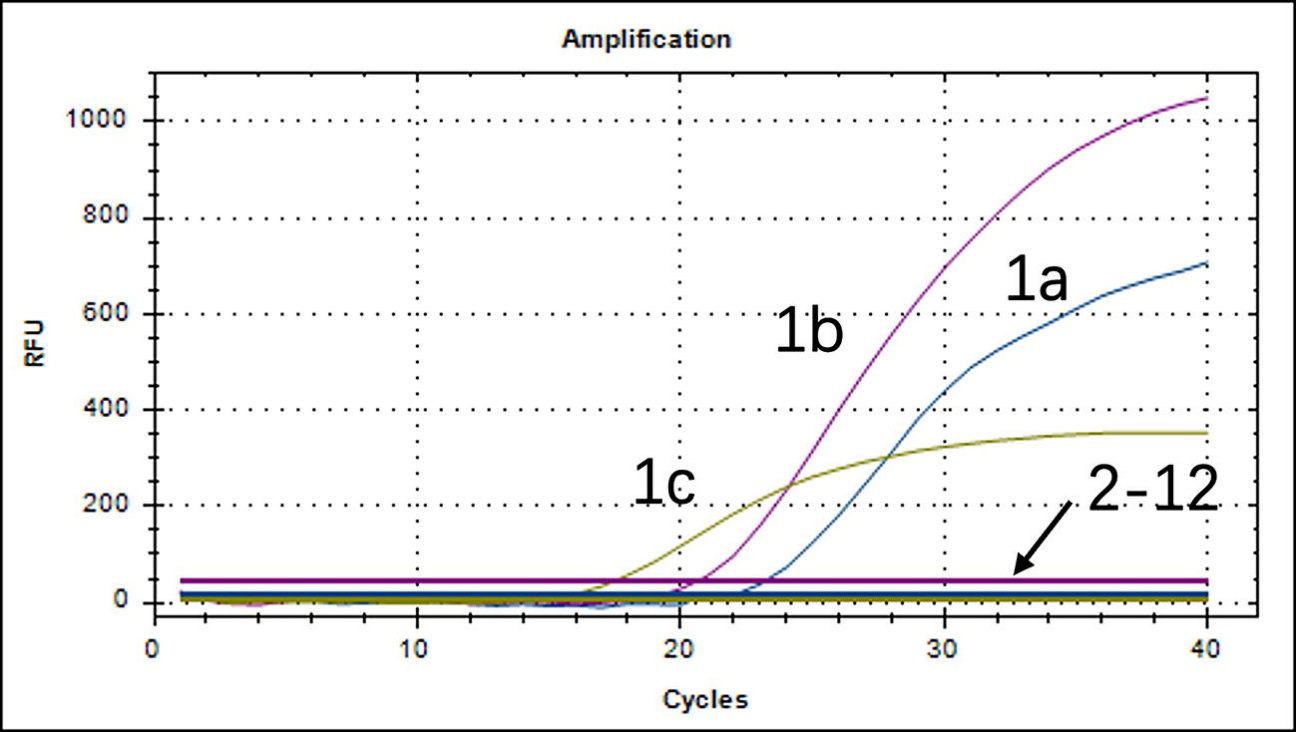
Amplification curves in the specificity test of the triplex real-time PCR assay. 1: Positive sample (1a: LI, 1b: Bhy, 1c: Cp); 2–12: *E. coli*, *Salmonella*, PEDV, TGEV, PDCoV, PoRV, Kp, PRV, PRRSV, PCV2, and negative control.

We chose different standard plasmids at ∼10^2^ copies/μL or ∼10^6^ copies/μL as the DNA templates to test the coefficient of variation (C.V.) values of the method. The results showed that C.V. values determined within different detection groups and between different detection groups were lower than 1.5% (Table 2), indicating the developed method possesses good stability. Specificity tests revealed that only DNA samples from LI, Bhy, and Cp showed positive amplification curves for the three fluorescence channels of FAM, Cy5, and VIC; while those from *Escherichia coli*, *Salmonella,* porcine epidemic diarrhea virus (PEDV), transmissible gastroenteritis virus of swine (TGEV), porcine delta coronavirus (PDCoV), porcine rotavirus (PoRV), *Klebsiella pneumoniae* (Kp), pseudorabies virus (PRV), porcine reproductive and respiratory syndrome virus (PRRSV), and porcine circovirus type 2 (PCV2) did not show amplification curves (Fig. 3). In addition, sensitivity tests showed that the detection limits of LI, Bhy, and Cp were 2.82, 14.1, and 5.63 copies/μL, respectively, by testing standard plasmids (10^−1^–10^9^) with different copy numbers (Table 3).

**TABLE 2:** Validation of the detection repeatability of the developed triplex real-time PCR method

Species	Copies/μL	Ct^*a*^ values of intra-assay		Ct values of inter-assay	
		𝒙 ®± s	CV^*b*^ (%)	𝒙 ®± s	CV (%)
LI	1 × 10^7^	22.23 ± 0.18	0.79	22.26 ± 0.29	1.32
	1 × 10^6^	25.86 ± 0.27	1.06	25.60 ± 0.36	1.40
	1 × 10^5^	28.83 ± 0.18	0.61	28.80 ± 0.25	0.86
Bhy	1 × 10^7^	22.78 ± 0.21	0.93	22.80 ± 0.44	1.94
	1 × 10^6^	25.97 ± 0.22	0.87	26.09 ± 0.40	1.55
	1 × 10^5^	29.35 ± 0.24	0.82	29.36 ± 0.40	1.36
Cp	1 × 10^7^	25.45 ± 0.11	0.44	25.43 ± 0.20	0.80
	1 × 10^6^	28.31 ± 0.49	1.73	28.80 ± 0.20	0.68
	1 × 10^5^	32.14 ± 0.14	0.44	32.14 ± 0.21	0.66

^*a*^ Ct, cycle threshold.

^*b*^ C.V., coefficient of variation.

**TABLE 3:** Detection limits of triplex real-time PCR methods detecting the different concentrations of those standard plasmids

Pathogens	LI		Bhy		Cp	
10-fold gradient dilutions	Concentrations (copies/μL)	Ct value	Concentrations (copies/μL)	Ct value	Concentrations (copies/μL)	Ct value
10^3^	2.82 × 10^3^	28.75	1.41 × 10^3^	28.94	5.63 × 10^3^	23.27
10^2^	2.82 × 10^2^	32.01	1.41 × 10^2^	32.54	5.63 × 10^2^	26.78
10^1^	2.82 × 10^1^	35.82	1.41 × 10^1^	35.80	5.63 × 10^1^	30.31
10^0^	2.82	39.20	1.41	NA^*a*^	5.63	33.63
10^−1^	2.82 × 10^−1^	NA	1.41 × 10^−1^	NA	5.63 × 10^−1^	NA

^*a*^ NA, no Ct value.

### Comparison of the detection results of different methods and application of the triplex real-time PCR method in clinical samples

To evaluate the accuracy of tr-PCR, genomic nucleic acids were extracted from 40 tissue samples (fecal swabs, ileum, and environmental swabs) suspected of LI, Bhy, and Cp infections collected clinically. Use the tr-PCR (the triplex real-time PCR method developed in this study) established in this study, C-PCR (a conventional PCR method in China), and R-PCR (a reported PCR method) to detect these samples. The results showed that the detection rates using the triple PCR method were 57.50% (23/40), 20.00% (8/40), and 32.50% (13/40), respectively. C-PCR exhibited detection rates of 57.50% (23/40), 17.50% (7/40), and 32.50% (13/40) for LI, Bhy, and Cp, respectively. R-PCR exhibited detection rates of 52.50% (21/40), 17.50% (7/40), and 30.00% (12/40) for LI, Bhy, and Cp. These results indicate that the tr-PCR developed in this study has a higher detection rate or similar detection results for pathogens compared to C-PCR and R-PCR (Table 4).

**TABLE 4:** Comparison results between the method and different detection methods in this study

Sample	Types	LI			Bhy			Cp		
		Tr-PCR	C-PCR^*a*^	R-PCR^*b*^	Tr-PCR	C-PCR^*c*^	R-PCR^*d*^	Tr-PCR	C-PCR^*e*^	R-PCR^*f*^
1	FS^*g*^	+	+	+	−	−	−	−	−	−
2	FS	+	+	+	−	−	−	−	−	−
3	FS	−	−	−	+	+	+	+	+	+
4	FS	+	+	−	−	−	−	−	−	−
5	FS	−	−	−	+	+	+	−	−	−
6	FS	−	−	−	−	−	−	+	+	+
7	FS	−	−	−	−	−	−	+	+	+
8	FS	+	+	+	−	−	−	+	+	+
9	FS	−	−	−	−	−	−	−	−	−
10	FS	+	+	+	−	−	−	−	−	−
11	FS	+	+	+	−	−	−	−	−	−
12	FS	+	+	+	+	+	+	−	−	−
13	FS	+	+	+	−	−	−	−	−	−
14	FS	−	−	−	−	−	−	−	−	−
15	FS	+	+	+	−	−	−	−	−	−
16	FS	−	−	−	−	−	−	+	+	+
17	FS	+	+	+	+	+	+	−	−	−
18	FS	−	−	−	−	−	−	−	−	−
19	FS	+	+	+	−	−	−	−	−	−
20	Ileum	−	−	−	−	−	−	+	+	+
21	Ileum	−	−	−	−	−	−	+	+	+
22	Ileum	−	−	−	−	−	−	−	−	−
23	Ileum	+	+	+	−	−	−	+	+	+
24	Ileum	+	+	+	−	−	−	−	−	−
25	Ileum	−	−	−	+	+	+	+	+	+
26	Ileum	+	+	+	+	−	−	−	−	−
27	Ileum	−	−	−	−	−	−	+	+	+
28	Ileum	+	+	+	−	−	−	+	+	+
29	Ileum	+	+	+	−	−	−	−	−	−
30	Ileum	+	+	+	−	−	−	−	−	−
31	Ileum	−	−	−	−	−	−	−	−	−
32	Ileum	+	+	+	−	−	−	+	+	+
33	Ileum	+	+	+	−	−	−	−	−	−
34	Ileum	−	−	−	+	+	+	−	−	−
35	Ileum	+	+	+	−	−	−	−	−	−
36	ES^*h*^	−	−	−	−	−	−	−	−	−
37	ES	+	+	−	+	+	+	−	−	−
38	ES	+	+	+	−	−	−	−	−	−
39	ES	+	+	+	−	−	−	−	−	−
40	ES	−	−	−	−	−	−	+	+	−
Positive control		+	+	+	+	+	+	+	+	+
Negative control		−	−	−	−	−	−	−	−	−

^*a*^ C-PCR, an industry standard PCR for the diagnosis of LI (SN/T 3844-2013).

^*b*^ R-PCR, a reported PCR methods for the diagnosis of LI (21).

^*c*^ C-PCR, an industry standard PCR for the diagnosis of Bhy (SN/T 1207-2011).

^*d*^ R-PCR, a reported PCR methods for the diagnosis of Bhy (22).

^*e*^ C-PCR, a national standard PCR for the diagnosis of Cp (GB 4789.13-2012).

^*f*^ R-PCR, a reported PCR methods for the diagnosis of Cp (23).

^*g*^ FS, fecal swab.

^*h*^ ES, environmental swab; “+” refers to be positive; “−” refers to be negative.

**Fig 4:**
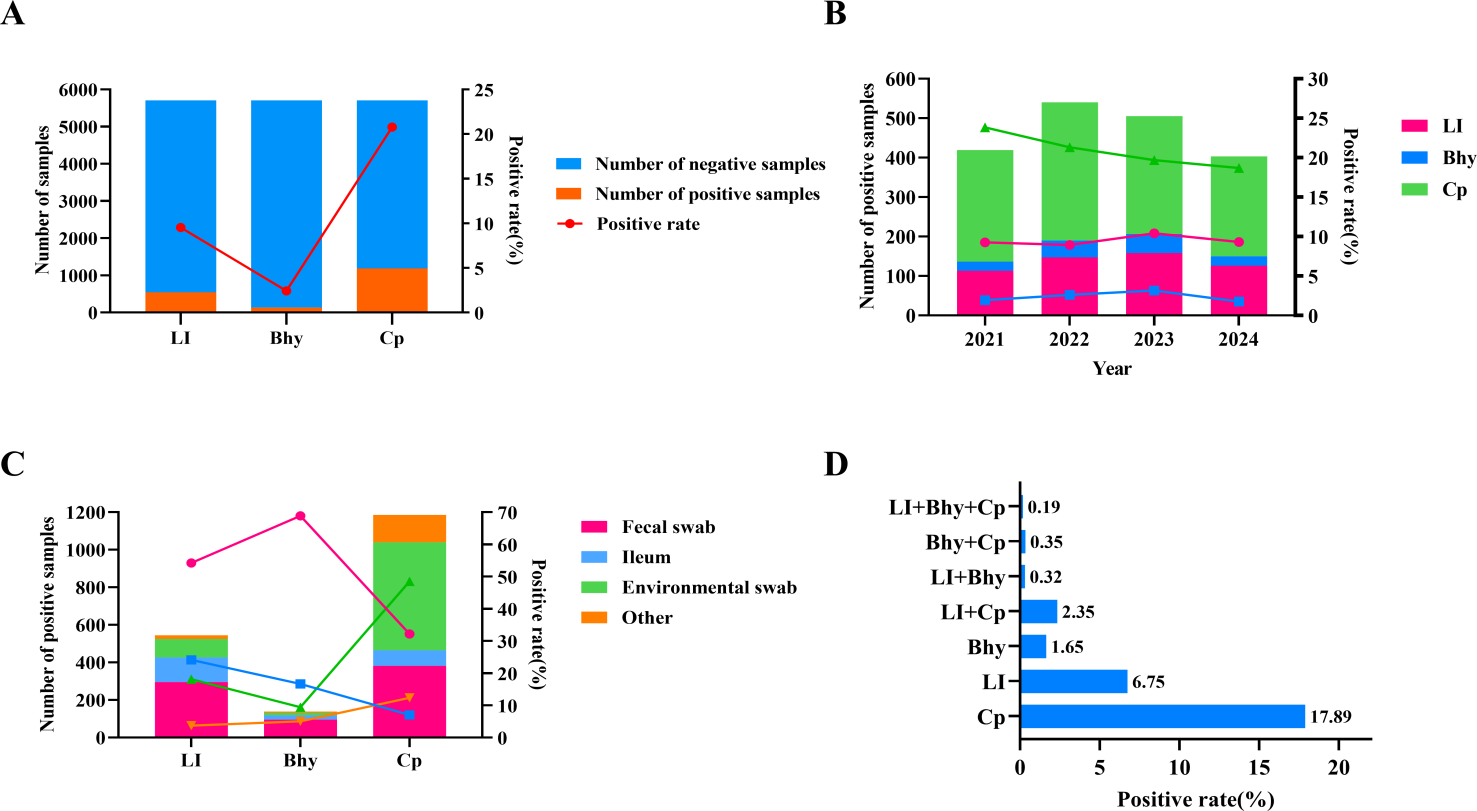
Investigation of clinical samples from pig farms and slaughterhouses in China using the triplex real-time PCR method. (**A**) Sample positivity of LI, Bhy, and Cp investigated using the triplex real-time PCR method; (**B**) positive rates of LI, Bhy, and Cp using the triplex real-time PCR method from 2021 to 2024; (**C**) the sources of positive samples for LI, Bhy, and Cp were determined using the triplex real-time PCR method; (**D**) detection of LI, Bhy, and Cp mixed infections using the triplex real-time PCR method.

From 2021 to 2024, 5,702 ileal samples from dead pigs, fecal swabs (fattening pigs and sows), and environmental swabs from pig farms and slaughterhouses that have never been vaccinated were collected, and LI, Bhy, and CP were detected by the triple qPCR method established in this study. The results showed that the overall detection rates of the three pathogens were 9.54% (544/5,702; LI), 2.42% (138/5,702; Bhy), and 20.78% (1,185/5,702; Cp) (Fig. 4A). According to the year, the positive detection rate trend was relatively stable, without significant difference, of which the highest positive detection rates of LI and Bhy were 10.41% (158/1,518) and 3.16% (48/1,518) in 2023, respectively, and the highest positive detection rate of Cp was 23.84% (283/1,187) in 2021 (Fig. 4B). This epidemic trend did not show significant fluctuations, but it also proved that there have been epidemics of the three pathogens, which should be paid attention to by pig farm breeders. According to the source of positive samples, LI and Bhy had the highest positive detection rates in fecal swabs (54.23%, 295/544; 68.84%, 95/138), respectively, and Cp had the highest positive detection rate in environmental swabs (48.52%, 575/1,185) (Fig. 4C). According to mixed infection, the single positive detection rates of LI, Bhy, and Cp were 6.75% (358/5,702), 1.65% (94/5,702), and 17.89% (1,020/5,702), respectively. In addition, some mixed infections were observed, including LI+Bhy 0.23% (13/5,702), LI+Cp 2.35% (134/5,702), Bhy+Cp 0.35% (20/5,702) and LI+Bhy+Cp 0.19% (11/5,702) (Fig. 4D). Although mixed infections are still rare at present, further monitoring is needed, and this situation cannot be ignored.

## DISCUSSION

Enteric bacterial infections are among the most common and economically significant diseases affecting swine production worldwide. Clinical signs of these infections include diarrhea, reduced growth rate, weight loss, and death of grower-finisher, young, and adult-age breeding animals (19). The most common bacterial pathogens include *L. intracellularis*, *B. hyodysenteriae*, *C. perfringens*, *E. coli*, and *Salmonella* (2, 24), and mixed infections are relatively common (6, 10). However, there have been no reports on rapid detection methods against *L. intracellularis*, *B. hyodysenteriae*, and *C. perfringens*, nor multiplex PCR detection methods. Routine bacterial diarrhea detection methods include bacterial isolation and identification, but this method cannot achieve rapid and accurate differential diagnosis of each pathogen (25, 26).

Due to the high sensitivity and ease of use of PCR and real-time PCR testing, several assays for bacterial diarrhea-related agents have been developed (10, 27, 28). Recently, Willems et al. reported a multiple qPCR detection method for *B. hyodysenteriae*, *Brachyspira pilosicoli*, and *L. intracellularis* in pig feces. The minimum number of copies detected is 10, 14, and 26 DNA copies, respectively (29). Compared to our method, the sensitivity was lower; LI is 2.4 copies/μL and Bhy is 14.1 copies/μL. La et al. developed a fast, sensitive, specific, and simultaneous detection method for three important intestinal bacterial pathogens in pig feces: *L. intracellularis*, *B. hyodysenteriae*, and *B. pilosicoli* (21). Compared with our method, the coincidence rate of clinical samples was lower, LI (21/23, 91.30%), Bhy (7/8, 87.5%).

The *aspA* (aspartate ammonia lyase) gene is a conserved gene in LI and already has a target gene used in PCR reactions (30–33). The *16S rRNA* gene is a unique marker of bacteria and is commonly used for species identification. *α-toxin A* toxin gene that can be used to identify toxin-producing *C. perfringens* (34). To optimize primers and probes, we confirmed the specificity of primers and probes by BLAST analysis at NCBI. In the reported literature, the optimal concentration of probe and primer, as well as annealing temperature, will also be optimized and selected, which is a necessary process to establish the qPCR method (35, 36). With the necessary screening, we identified parameters that could yield the best amplification results, with optimal concentrations of 0.25 and 0.5 µM for primers and probes, respectively, and an annealing temperature of 60°C. These parameters ensure high amplification efficiency of the reaction (Fig. 2).

Specificity and sensitivity are important indicators for measuring the accuracy of diagnostic testing (35). The maximum intra-group coefficient of variation of this method was 1.73% and the inter-group coefficient of variation was 1.94%, indicating good stability of this method (Table 2). As shown in Fig. 2, standard plasmids (10^8^–10^1^) tested for different copy numbers had regression coefficients (*R^2^*) greater than 0.99, good correlation, and high sensitivity of primers and probes. The lowest limit of LI, Bhy, and Cp detected by this method was 2.82, 14.1, and 5.63 copies/μL, respectively. Compared to the reported literature, the method has a lower limit of detection (LI, 2.82 < 36 copies/μL) (37). When three positive pathogens were amplified simultaneously, the primers and probes in the amplification curves did not generate cross-reactivity between pathogens or non-specific reactivity with other pathogens (Fig. 3). In the previously reported methods, the repeatability and specificity of the method were not mentioned, and the sensitivity was worse than that of the method in this study (10, 21). In this study, our assay can simultaneously detect LI, Bhy, and CP and can quickly distinguish and diagnose three pathogens under the same system, improving work efficiency and saving 2/3 of the time and cost.

Compared with the published traditional detection methods (industry standards, national standards) and the methods established in the reported studies, the detection results of this method were superior or similar to those of suspicious samples collected from clinical sources (ileum, fecal swab, environmental swab), especially the detection rate of LI was better than that of R-PCR, and the detection rate of Bhy was better than that of tr-PCR and R-PCR (21–23).

Mixed bacterial infections are common in swine production, and many clinical symptoms are still very similar, urgently requiring rapid high-throughput diagnostic methods to identify syndromic pathogens (38, 39). In addition, with increasing international trade, animal transport, and human travel, rapid diagnosis is essential to prevent the widespread spread of animal diseases (20, 40). From 2021 to 2024, we investigated 5,702 samples from pig farms and slaughterhouses in China, in which the positive detection rate of LI was 9.54% (544/5,702). This result is also similar to the positive rate of a recent epidemiological study performed in six European countries by detecting *L. intracellularis* DNA from fecal samples from diarrheal pigs using qPCR (26.2%) (41). It was also similar to the detection of *B. hyodysenteriae* (7.9%) and *L. intracellularis* (3.6%) in pig feces (29). LI, Bhy, and Cp could also be detected in environmental samples in this study, suggesting that the environment is also an important route of transmission for bacterial diarrhea, and environmental swabs have generally not been tested in previous studies, a route that is easily overlooked. Cp is an opportunistic pathogen that can form extremely resistant spores, which can germinate and grow under suitable conditions commonly encountered in the environment, and can become bacteria when the appropriate temperature is reached (42, 43). The method of this study combined with the clinical symptoms of pigs on the farm can be used for comprehensive judgment to achieve early warning and rapid diagnosis. Using this method, clinical samples were tested, suggesting that the three pathogens were mainly infected alone, and mixed infections also emerged, which was similar to previous reports (16, 23, 29, 44). In the current research on the pathogen of diarrhea in pig farms, we often mainly focus on the mixed infection between viruses (45, 46), and bacterial infection is often not prioritized, suggesting that we should pay attention to the mixed infection of bacterial diarrhea such as LI, Bhy, and CP in the future detection of animal diseases in pig farms. Recent studies demonstrate that Cp co-infections with *L. intracellularis* or *B. hyodysenteriae* exacerbate enteric lesions, even in post-weaned and growing pigs (3, 6, 7, 9), which manifests as blood dysentery, abdominal flatulence, and acute death after infection, and its harm cannot be ignored.

This triplex real-time PCR method comprehensively and simultaneously detects multiple bacterial diarrhea pathogens (LI, Bhy, and CP) and rapidly confirms their presence in samples. Using this method, we investigated the distribution of these three bacterial diarrhea-associated pathogens in pig farms and slaughterhouses in China, and the results showed that the pathogen of bacterial diarrhea should not be ignored, and farms need to be concerned.

## MATERIALS AND METHODS

### Primer and probe design

Target gene sequences were obtained through a BLAST search on the GenBank website. The primers and probes were designed in the most conserved region of the target gene (*aspA, 16s rRNA, α-toxin*) that was identified from multiple sequence alignments to cover as many sequences as possible. Primers and probes targeting genes were designed using the SnapGene software (Version 5.3; https://www.snapgene.com/) and the Primer Premier 5 program. The probe for LI was labeled with the 5′-reported dye 6-carboxyfluorescein (FAM) and the 3′-quencher BHQ1, the probe for Bhy was labeled with the 5′-reported dye Cy5 and the 3′-quencher BHQ2, and the probe for Cp was labeled with the dye VIC and the 3′-quencher BHQ1 (Table 1).

### Optimization of PCR reaction system and conditions

Genomic DNA was extracted using a Vazyme DNA/RNA Extraction Kit (Cat No. RM-201-02; Nanjing, China) following the manufacturer’s instructions. The triplex real-time PCR assay was performed in a 20.0 µL reaction volume, which contains template DNA 4.0 µL, AceQR Universal U+ Probe Master Mix (Vazyme, Nanjing, China) 10.0 µL, each of the forward and reverse primers (0.20, 0.25, 0.30, 0.35, 0.40, 0.45, 0.50, or 0.55 µM), each of the TaqMan probes (0.25, 0.30, 0.35, 0.40, 0.45, or 0.50 µM), and nuclease-free water up to 20.0 µL. A PCR assay was performed on a CFX96 Touch Real-Time PCR Detection System (Bio-Rad, Hercules, CA) with the following conditions: 95°C for 5 min, followed by 40 cycles of 95°C for 15 s, annealing at different temperatures (55–62°C) for 45 s, and the fluorescence signal was recorded. The copy number of the recombinant plasmids was calculated using the following formula: copy number (copies/μL) = NA (copies/mol) × concentration (g/μL)/MW (g/mol), where NA is Avogadro’s number and MW is the base number times 340.

### Construction of standard curves

The partial length of LI (588 bp, base pairs 160–747), Bhy (600 bp, base pairs 2–601), and Cp (560 bp, base pairs 3227–3786) from the whole genome sequence of LI N343 (GenBank accession no. CP004029.1), partial sequence of Bhy strain B78 16S ribosomal RNA (GenBank accession no. NR_044764.1), and whole genome sequence of Cp CPI 18-6 (GenBank accession no. CP075979.1) were synthesized and cloned into the pUC-57 vector (TaKaRa, China) to obtain the recombinant plasmids pUC57-LI, pUC57-Bhy, and pUC57-Cp. According to the manufacturer’s protocol for plasmid resuspension, these recombinant plasmids were centrifuged and re-suspended in 40 µL of nuclease-free water and then stored at −20°C before use.

To generate standard curves, a series of 10-fold dilutions (10^−2^–10^−9^) were given to the three synthesized standard plasmids (pUC57-LI, pUC57-Bhy, and pUC57-Cp), which were used as the template DNA to perform the triplex real-time PCR assays. Standard curves were generated based on the Ct values and the copy numbers (lg values) of the template DNA. Coefficients of determination (*R^2^*) were calculated using GraphPad Prism v. 8.0.1. The reaction efficiency is calculated using the following formula: E = 10^(−1/k)−1 (k represents the slope of the standard curve).

### Validation of specificity, sensitivity, and stability

The specificity of the generated triplex real-time PCR method was validated using the genomic DNA extracted from the other pathogens, including *Escherichia coli*, *Salmonella*, PEDV, TGEV, PDCoV, PoRV, Kp, PRV, PRRSV, and PCV2.

To assess the sensitivity of the triplex real-time PCR method, Ct values were determined by testing different copy numbers (10^−1^–10^9^) of recombinant standard plasmids pUC57-LI, pUC57-Bhy, and pUC57-Cp. The sensitivity of the method was determined based on the lowest detectable copy number.

To test the stability of the generated triplex real-time PCR method, three different concentrations of standard plasmids pUC57-LI, pUC57-Bhy, and pUC57-Cp (10^5^, 10^6^, 10^7^ copies/μL) were used as templates. Three batches were tested using the quantitative PCR method, and each sample was performed in triplicate. Ct values were compared for different concentrations of recombinant standard plasmids, and intra-group and inter-group repeatability results were used to calculate each coefficient of variation.

### Comparison with general methods

We also compared the detection results of the triplex real-time PCR method developed in this study (hereinafter referred to as the “tr-PCR”), a conventional PCR method in China (hereinafter referred to as the “C-PCR”) and a reported PCR method (hereinafter referred to as the “R-PCR”) (21–23). DNA was extracted from 40 different types of samples collected from different pig farms (pig manure, pig tissue, environmental swabs) and detected using a triple real-time PCR method. All the samples were transported at 4°C and kept at −80°C for long-term storage. Genomic DNA was extracted using the magnetic bead-based DNA/RNA Extraction Kit (Vazyme, Nanjing, China) following the manufacturer’s instructions. C-PCR and R-PCR assays were conducted in a 20.0 µL reaction system, which contained 10.0 µL of 2× Taq Master Mix (Vazyme Biotech, China), 0.5 µM of the primers for, respectively, 1.0 µL of template, and ddH_2_O to a final volume of 20.0 µL. The amplification program was that pre-denaturation at 95°C for 5 min, followed by 40 cycles of sequential denaturation at 95°C for 30 s, annealing at 54°C for 30 s and, extension at 72°C for 20 s, a final extension at 72°C for 10 min. The PCR products were analyzed by 1.5% agarose gel electrophoresis. ddH_2_O was used as a negative control.

### Clinical sample investigation

Sample detection using the triple quantitative real-time PCR method from 2021 to 2024, a total of 5,702 fecal swabs (fattening pigs and sows), environmental swabs, and ileum were collected from various pig farms in China. Samples were treated as follows: (i) fecal samples were mixed thoroughly with an equal volume of physiological saline, followed by centrifugation at 2,000 rpm for 2 min to collect the supernatants; (ii) swabs were maintained in tubes containing 500 µL of physiological saline and shaken vigorously, followed by centrifugation at 2,000 rpm for 2 min to collect the supernatants; (iii) intestinal samples (50–100 mg) were homogenized in 1 mL of physiological saline, followed by centrifugation at 2,000 rpm for 2 min to collect the supernatants. Afterward, DNAs were extracted using a DNA/RNA Extraction Kit (Vazyme, Nanjing, China) according to the manufacturer’s instructions, using the method established in this study for detection.
